# Neoadjuvant plus adjuvant or only adjuvant nab-paclitaxel plus gemcitabine for resectable pancreatic cancer - the NEONAX trial (AIO-PAK-0313), a prospective, randomized, controlled, phase II study of the AIO pancreatic cancer group

**DOI:** 10.1186/s12885-018-5183-y

**Published:** 2018-12-29

**Authors:** Thomas J. Ettrich, Andreas W. Berger, Lukas Perkhofer, Severin Daum, Alexander König, Andreas Dickhut, Uwe Wittel, Kai Wille, Michael Geissler, Hana Algül, Eike Gallmeier, Jens Atzpodien, Marko Kornmann, Rainer Muche, Nicole Prasnikar, Andrea Tannapfel, Anke Reinacher-Schick, Waldemar Uhl, Thomas Seufferlein

**Affiliations:** 1 Department of Internal Medicine I, University of Ulm, Albert-Einstein-Allee 23, 89081 Ulm, Germany; 2 Department of Gastroenterology, Infectious Diseases and Rheumatology, Charité University Medicine Berlin, Hindenburgdamm 30, 12200 Berlin, Germany; 3 Department of Gastroenterology and Gastrointestinal Oncology, University Medical Center Goettingen, Robert-Koch-Str. 40, 37075 Göttingen, Germany; 4 Department of Oncology/Hematology, Fulda Hospital, Pacelliallee 4, 36043 Fulda, Germany; 5 Department of General and Visceral Surgery, University of Freiburg, Hugstetter Str. 55, 79106 Freiburg, Germany; 6 Department of Hematology and medical oncology, Johannes-Wesling-Klinikum Minden, Hans-Nolte-Straße 1, 32429 Minden, Germany; 7 Department of Internal Medicine, Oncology/Hematology, Gastroenterology, Esslingen Hospital, Hirschlandstr. 97, 73730 Esslingen, Esslingen, Germany; 8 Department of Internal Medicine II, Technical University Munich, Ismaninger Str. 22, 81675 Munich, Germany; 9 Department of Gastroenterology and Endocrinology, University of Marburg, Baldingerstraße, 35043 Marburg, Germany; 10 Department of Medical Oncology and Hematology, Niels-Stensen-Kliniken, Alte Rothenfelder Str. 23, 49124 Georgsmarienhütte, Germany; 11 Department of General and Visceral Surgery, University of Ulm, Albert-Einstein-Allee 23, 89081 Ulm, Germany; 12 Institute of Epidemiology and Medical Biometry, University of Ulm, Schwabstrasse 13, 89081 Ulm, Germany; 13 Department of Oncologie, Asklepios Klinik Barmbek, Rübenkamp 220, 22291 Hamburg, Germany; 14 Department of Pathology, Ruhr-University Bochum, Bürkle-de-la-Camp-Platz 1, 44789 Bochum, Germany; 15 Department of Internal Medicine, Ruhr-University Bochum, Gudrunstr. 56, 44791 Bochum, Germany; 16 Department of Surgery, Ruhr-University Bochum, Gudrunstr. 56, 44791 Bochum, Germany

**Keywords:** Pancreatic ductal adenocarcinoma, Pancreatic cancer, Resectable, Neoadjuvant chemotherapy, Perioperative chemotherapy

## Abstract

**Background:**

Even clearly resectable pancreatic cancer still has an unfavorable prognosis. Neoadjuvant or perioperative therapies might improve the prognosis of these patients. Thus, evaluation of perioperative chemotherapy in resectable pancreatic cancer in a prospective, randomized trial is warranted. A substantial improvement in overall survival of patients with metastatic pancreatic cancer with FOLFIRINOX and nab-paclitaxel/gemcitabine vs standard gemcitabine has been demonstrated in phase III-trials. Indeed nab-paclitaxel/gemcitabine has a more favorable toxicity profile compared to the FOLFIRINOX protocol and appears applicable in a perioperative setting.

**Methods:**

NEONAX is an interventional, prospective, randomized, controlled, open label, two sided phase II study with an unconnected analysis of the results in both experimental arms against a fixed survival probability (38% at 18 months with adjuvant gemcitabine), NCT02047513. NEONAX will enroll 166 patients with resectable pancreatic ductal adenocarcinoma (≤ cT3, N0 or N1, cM0) in two arms: Arm A (perioperative arm): 2 cycles nab-paclitaxel (125 mg/m2)/gemcitabine (1000 mg/m2, d1, 8 and 15 of an 28 day-cycle) followed by tumor surgery followed by 4 cycles nab-paclitaxel/gemcitabine, Arm B (adjuvant arm): tumor surgery followed by 6 cycles nab-paclitaxel/gemcitabine. The randomization (1:1) is eminent to avoid allocation bias between the groups. Randomization is stratified for tumor stage (ct1/2 vs. cT3) and lymph node status (cN0 vs. cN1). Primary objective is disease free survival (DFS) at 18 months after randomization. Key secondary objectives are 3-year overall survival (OS) rate and DFS rate, progression during neoadjuvant therapy, R0 and R1 resection rate, quality of life and correlation of DFS, OS and tumor regression with pharmacogenomic markers, tumor biomarkers and molecular analyses (ctDNA, transcriptome, miRNA-arrays). In addition, circulating tumor-DNA will be analyzed in patients with the best and the worst responses to the neoadjuvant treatment. The study was initiated in March 2015 in 26 centers for pancreatic surgery in Germany.

**Discussion:**

The NEONAX trial is an innovative study on resectable pancreatic cancer and currently one of the largest trials in this field of research. It addresses the question of the role of intensified perioperative treatment with nab-paclitaxel plus gemcitabine in resectable pancreatic cancers to improve disease-free survival and offers a unique potential for translational research.

**Trial registration:**

ClinicalTrials.gov: NCT02047513, 08/13/2014.

**Electronic supplementary material:**

The online version of this article (10.1186/s12885-018-5183-y) contains supplementary material, which is available to authorized users.

## Background

Pancreatic ductal adenocarcinoma (PDAC) is still one of the most lethal cancers in the Western world [1]. Whereas overall survival of several solid tumors, e.g. colorectal cancer, has constantly and substantially improved over the recent years, only minor progress has been made in PDAC [2]. Moreover, forecasts predict only a marginal improvement in overall survival by 2030 when pancreatic cancer will be the second leading cause of cancer related deaths [3].

The only curative approach for PDAC is surgery. However, only 15–20% of the patients are definitely eligible for surgery in curative intent at the time of primary diagnosis. Nevertheless, there is evidence from computational modeling analyses that the majority of pancreatic cancers are primarily metastatic even if deemed resectable [4]. R0 resection, followed by adjuvant chemotherapy confers the best prognosis and therefore is the state of the art therapy [5–7]. However, with this therapeutic approach median overall survival (mOS) times of at best 28 months and 5-year overall survival rates around 29% are possible in a Western population [7]. Although pancreatic surgery has improved with respect to morbidity and perioperative mortality during this time, substantial improvement in overall survival remains lacking as indicated by results from randomized trials. So the main improvement over the last decades was achieved by adjuvant chemotherapy, considering that there is an urgent need for novel strategies. Neoadjuvant or perioperative therapies have proven to be successful in improving overall survival in other solid gastrointestinal cancers such as gastric or esophageal cancer [8]. Thus, this strategy may also be beneficial to improve the prognosis of at least some patients with PDAC by inducing tumor shrinkage/downsizing and/or preventing metastasis.

### Evidence

R0 surgical resection is the only curative treatment for pancreatic cancer, but the R0 resection rate can be as low as 20% due to the early invasion of the tumor. “R0” requires accurate pathological assessment and there are shortcomings in assessing the retropancreatic area and the vessel plane to exactly differentiate between an R0 and R1 status [9–11]. R1 resections are consistently associated with worse outcome. An efficient downsizing/downstaging of the tumor preoperatively by neoadjuvant treatment could improve the R0 resection rate and potentially overall survival of patients with resectable PDAC. However, even after R0 resection, the relapse rate of pancreatic cancer is high due to both local recurrence (50–75%) and distant metastasis (liver 60–90%, peritoneum 40%), particularly in T3/T4 tumors [12]. Adjuvant chemotherapy with 5-FU, gemcitabine or the combination of gemcitabine and capecitabine is the only established standard in resectable pancreatic cancer and increases the 5-year survival rate in clinical trials from 8% up to 28% [6, 7, 12]. It is unclear whether protocols that are more efficient in the metastatic situation such as FOLFIRINOX or nab-paclitaxel plus gemcitabine confer additional benefit in this situation.

Currently the use of “neoadjuvant” chemotherapy or radiochemotherapy is only accepted in case of borderline resectability and/or locally advanced disease although the available evidence for this strategy is also sparse [13, 14]. This is due to a low efficacy of previously used chemotherapy or radiochemotherapy regimens with response rates below 10% in case of gemcitabine. The extensive desmoplastic stroma in pancreatic cancer also prevents proper tumor penetration by chemotherapy and we still do not have a standardized radiochemotherapy protocol with proven efficacy in controlled trials for this situation. Since borderline resectability and locally advanced disease are also not defined consistently, in many studies the patient population is mixed and consequently, data on the efficacy of neoadjuvant radiochemotherapy or chemotherapy are varying with median overall survival times ranging from 10 to 36 months [15–18]. Most of the trials on neoadjuvant treatment did not examine the degree of tumor downsizing by the respective protocol and did not establish a correlation of tumor downsizing with outcome parameters (R0 resection rate, disease free survival (DFS) or OS).

Recently, two phase III trials demonstrated, for the first time, a substantial improvement in PFS and OS in patients with metastatic pancreatic cancer compared to standard gemcitabine. These trials used either a combination of 5-FU, irinotecan and oxaliplatin (FOLFIRINOX protocol; mPFS 6.4 vs. 3.3 months, mOS 11.1 vs. 6.8 months, always compared to gemcitabine monotherapy) or the combination of nab-paclitaxel plus gemcitabine (mPFS 5.5 vs. 3.7 months, mOS 8.7 vs. 6.6 months compared to gemcitabine alone) [19, 20]. These data demonstrate that these combinations can overcome chemotherapy-resistance of metastatic pancreatic cancer. Tumor response was remarkable with a 31% response rate (RR) in the FOLFIRINOX group and a 29% RR in the nab-Paclitaxel/gemcitabine group compared to 7 and 9.4% RR, respectively, with gemcitabine alone [20, 21]. Thus, these two regimens have obvious advantages compared to the commonly used chemotherapeutic regimens in pancreatic cancer and therefore also appear as promising regimens in the neoadjuvant setting [21]. However, the FOLFIRINOX protocol is associated with a substantial rate of grade 3/4 neutropenia (45.7%) and diarrhea (12.7%). 42% of the patients in the FOLFIRINOX trial received G-CSF [22]. The combination of nab-paclitaxel/gemcitabine is more toxic compared to gemcitabine alone (38% grade 3/4 neutropenia compared to 27%), but its toxicity profile seems to be more favorable compared to the FOLFIRINOX protocol.

There are only few data on the efficacy of this treatment in a neoadjuvant, perioperative or adjuvant treatment setting. However, a recently published meta-analysis for the use of FOLFIRINOX for locally advanced PDAC nicely showed a prolonged mOS of 24.2 months longer in patients treated with the FOLFIRNOX regimen compared to gemcitabine [23]. A recent study using nab-paclitaxel plus gemcitabine in resectable PDAC showed a resection rate of 75% and an R0 resection rate of 92% [24]. Similar data were reported by other groups [25, 26]. This in line indicates that these protocols are also beneficial for non-metastatic PDAC.

#### Rationale for the trial

The high efficacy and good tolerability makes the combination of nab-Paclitaxel and gemcitabine an interesting regimen for neoadjuvant therapy to be examined. In the NEONAX trial we will determine the impact of 2 cycles of neoadjuvant nab-paclitaxel/gemcitabine followed by surgery and 4 cycles of adjuvant nab-paclitaxel/gemcitabine or 6 cycles of adjuvant nab-paclitaxel/gemcitabine on the DFS rate at 18 months post randomization. Our aim is to increase the DFS rate at 18 months from 38% as described for gemcitabine [6] to ≥55% in at least one of the experimental arms. Noteworthy, the trial is not statistically powered to compare efficacy between both therapeutic strategies.

The rationale for using two cycles of neoadjuvant chemotherapy in the perioperative treatment group is based on the findings of a phase I trial. There has been an obvious difference in the tolerability with increasing therapy cycles, thereby two cycles of nab-paclitaxel plus gemcitabine were tolerated by most of the patients whereas only about 60% of the patients could receive three cycles. Furthermore, this treatment was effective with grade 3–4 tumor regression in 30% of the tumors [27]. Additionally, a significant decrease in FDG-uptake was observed in a phase I/II trial already after 6 weeks of treatment with nab-paclitaxel/gemcitabine suggesting that 2 cycles of this regimen are efficacious in the neoadjuvant setting [28]. Finally, PDAC has still a dismal prognosis and further delays in surgery may not be acceptable to patients and therefore decrease compliance with the protocol, which was not seen after 2 cycles of nab-paclitaxel/gemcitabine.

The doses of nab-paclitaxel and gemcitabine proposed for this trial are derived from the palliative use of this combination. The time frame for the adjuvant treatment (within 12 weeks after surgery) is derived from previous adjuvant trials in PDAC and reflects the fact that patients still benefit from adjuvant chemotherapy when the treatment starts within 12 weeks after surgery but all cycles of chemotherapy are given. Thus, completing all cycles seems to be more important than a very early start of chemotherapy after surgery [29].

## Methods and design

**Fig. 1:**
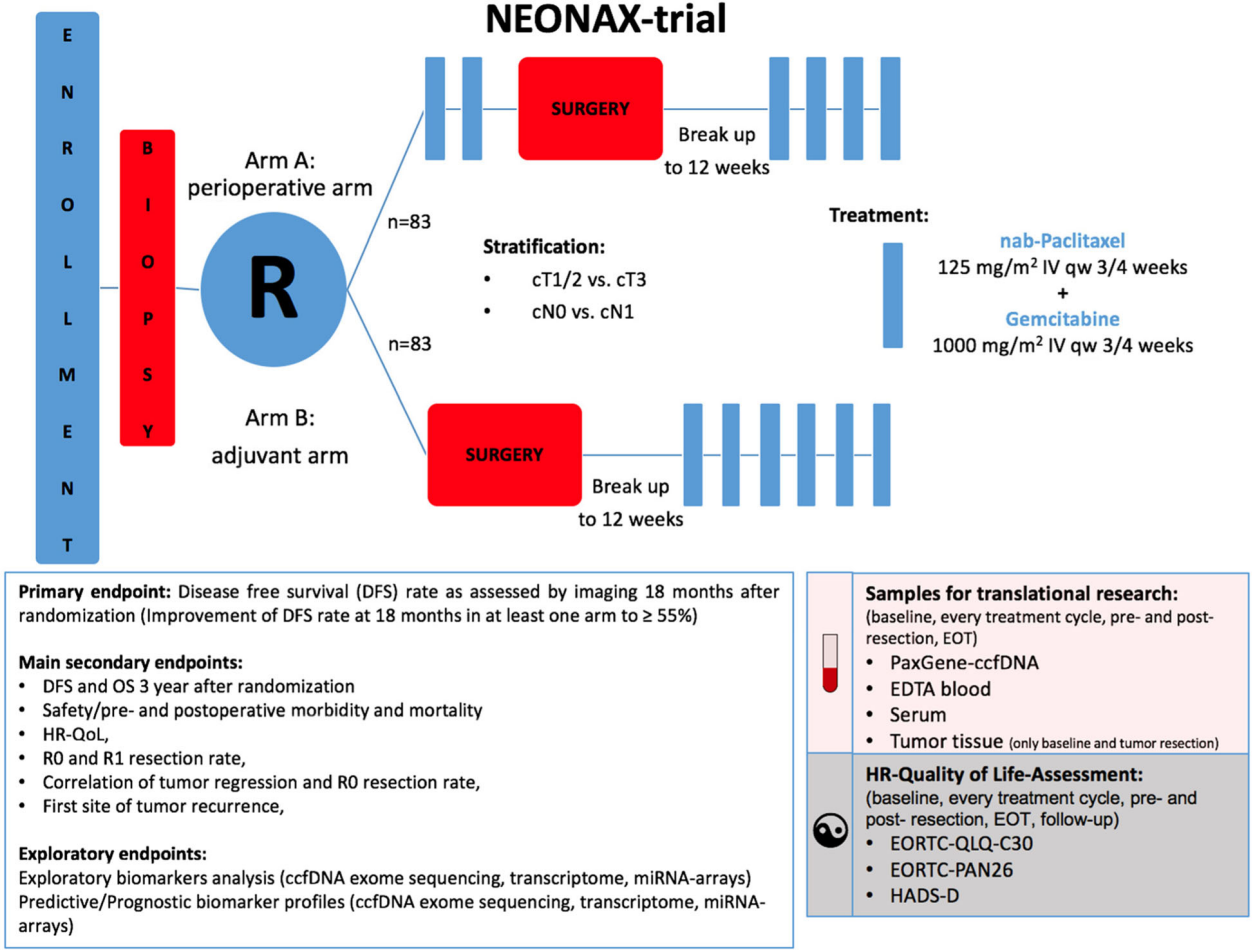
NEONAX-trial: flow-chart

NEONAX is an interventional, multi-center, prospective, randomized-controlled-trial. It is planned as a two sided, open label phase II study controlled against a fixed survival probability in an unconnected analysis of both experimental arms (see Fig. 1  Additional file 1).

### Study objectives

#### Primary objective

- Disease free survival (DFS) rate at 18 months post randomization (DFS rate improvement in one arm of at least ≥55%)

#### Secondary objectives

- To assess the effect of neoadjuvant nab-paclitaxel/gemcitabine on tumor response rate (RECIST 1.1), histological tumor regression and R0 resection rate
- Effects of perioperative or adjuvant nab-paclitaxel/gemcitabine on 3-years DFS and OS
- Safety
- Pre- and postoperative morbidity and mortality
- Toxicity assessment
- Disease progression rate under neoadjuvant nab-paclitaxel/gemcitabine
- R0 and R1 resection rates
- Correlation of tumor regression and R0 resection rate in the perioperative study arm
- Overall survival (OS)
- First site of tumor recurrence
- Health related quality of life (EORTC QLQ-PAN26, QLQ-C30 and HADS-D questionnaires)
- Correlation of DFS, OS and tumor regression with pharmacogenomic markers, tumor biomarkers and molecular analyses (ctDNA, transcriptome, miRNA-arrays)

### Patient selection and randomization

In total 166 have to be allocated to the trial and 116 have to be analyzed (58 per arm). This will be achieved by screening 190 patients in 30 planned sites. Randomization is 1:1 to the perioperative arm (arm A) or the adjuvant arm (arm B). Randomization strata are as follows: cT1/2 vs. cT3 and cN0 vs. cN1. For eligibility criteria see Table 1.

**Table 1:** Eligibility criteria for the NEONAX-trial

**Inclusion criteria:**	
• Histologically or cytologically proven clearly resectable ductal adenocarcinoma of the pancreas (PDAC) ≤ cT3 with no prior tumor specific treatment. (After consolidation with the coordinating investigator a cytological determination is possible in exceptional cases.)	
• No evidence of metastases to distant organs (e.g. liver, peritoneum, lung).	
• Resectable tumor. Determination of resectability based on spiral CT scans with both oral and i.v. contrast enhancement or on MRI using a recent consensus definition (Resectability: Clear fat planes around the celiac artery, hepatic artery and superior mesenteric artery. [5, 31])	
• ECOG performance status 0 or 1	
• Creatinine clearance ≥30 ml/min	
• Serum total bilirubin level ≤ 2.5 x ULN	
• ALT and AST ≤ 2.5 x ULN	
• In case of biliary obstruction, biliary decompression is required. Post-interventional bilirubin levels must be ≤2.5 x ULN	
• White blood cell count ≥3.5 × 10^6^/ml, neutrophil granulocytes count ≥1,5 × 10^6^/ml, platelet count ≥100 × 10^6^/ml	
• Signed informed consent incl. Participation in translational research	
• Age ≥ 18 years and < 75 years	
**Main exclusion criteria**	
• Borderline resectable PDAC by radiologic criteria, papillary cancer on neuroendocrine Cancer	
• Radiographic evidence of severe portal hypertension/cavernous transformation	
• Chronic infectious diseases, immune deficiency syndromes	
• Premalignant hematologic disorders, e.g. myelodysplastic syndrome	
• Disability to understand and sign written informed consent document	
• Past or current history of malignancies except for the indication under this study and curatively treated:	
▪ Basal and squamous cell carcinoma of the skin	
▪ In-situ carcinoma of the cervix	
▪ Other malignant disease without recurrence after at least 5 years of follow-up	
• Clinically significant cardiovascular disease (incl. Myocardial infarction, unstable angina, symptomatic congestive heart failure, serious uncontrolled cardiac arrhythmia) 6 months before enrollment	
• Clinically relevant or history of interstitial lung disease, e.g. non-infectious pneumonitis or pulmonary fibrosis or evidence of interstitial lung disease on baseline chest CT scan or chest x-ray.	
• Pre-existing neuropathy > grade 1 (NCI CTCAE)	
• Pregnancy or breastfeeding women.	

### Staging assessments (Additional file 2)

- Completed medical history and physical examination
- 12 lead ECG/echocardiography
- Contrast enhanced multislice CT of the abdomen/abdominal MRI and chest x-ray/thoracic CT, Ultrasound elasticity imaging of the tumor (optional)
- Hematological tests, Clinical chemistry
- Tumor Marker (Serum): Ca 19–9, CEA
- Signed written informed consent.
- PDAC diagnosis: Core biopsies of the tumor can be obtained via endoscopic ultrasound for histological or cytological assessment. Alternatively tissue samples can be obtained via laparoscopic surgery
- EORTC QLQ-PAN26, QLQ-C30 and HADS-D questionnaire

### Treatment

#### Arm A (perioperative arm)

Initial treatment with 2 cycles of nab-paclitaxel/gemcitabine (nab-paclitaxel 125 mg/m^2^, gemcitabine 1000 mg/m^2^ on day 1, 8 and 15 of a 28 day-cycle) followed by 3 weeks of rest and subsequent tumor surgery. Re-Start of chemotherapy within 12 weeks after surgery with in total 4 more cycles of nab-paclitaxel/gemcitabine in the adjuvant setting.

#### Arm B (adjuvant arm)

Tumor surgery followed by adjuvant chemotherapy with 6 cycles of nab-paclitaxel/gemcitabine (nab-Paclitaxel 125 mg/m^2^, Gemcitabine 1000 mg/m^2^ on day 1, 8 and 15 of a 28 day-cycle) starting within 12 weeks after surgery.

#### Surgery

Resectability is determined by contrast enhanced spiral CT or MRI and based on a recent consensus definition that determines resectability by a visualizable fat plane around celiac and superior mesenteric arteries and patent superior mesenteric/portal vein [30]. Surgery is performed according to the standards of the respective institution. A time interval of about 2–3 weeks between the last application of neoadjuvant chemotherapy and surgery is recommended.

### Follow-up

Follow-up documentation every 12 weeks until unequivocal detection of a relapse is mainly performed in order to assess the efficacy objectives of disease-free survival and overall survival. Staging procedures are to be documented up to 3 years after end of treatment.

- Physical examination including: weight, WHO/ECOG performance status
- EORTC QLQ-PAN26, QLQ-C30 and HADS-D questionnaire
- Abdominal CT/MRI and chest x-ray routinely every three months for 3 years, then abdominal ultrasound every 3 months (if suspicious for relapse: CT/MRI) and abdominal CT/MRI and chest x-ray every 6 months, as an alternative to chest -ray, thoracic CT can be performed at the discretion of the center (recommended)
- Additional Clinical tumor assessments, if appropriate
- Tumor marker (serum): Ca 19–9, CEA
- Eventual second / further line treatment
- Survival status

### Sample size calculation and statistical analysis

According to published literature DFS after adjuvant Gemcitabine at 18 months is about 38% [6]. To accept the novel combination as clinically relevant it should achieve at least an increase in the DFS rate at 18 months from 38% to at least 55%. An expected increase in the DFS rate at 18 months to 55% can be found with a power of 90% and a two-sided significance level of 5% with a one-sample logrank-test if 58 patients per treatment group (116 in total) are included in the study. Sample size was computed by “SWOG one arm survival sample size and power” based on Lawless [31]. This calculation assumes exponential survival, an accrual time of 36 months and a total observation time of 57 months. A 15% dropout rate is expected due to toxic side effects of nab-paclitaxel/gemcitabine, disease progress during the neoadjuvant treatment, intraoperative evidence of distant metastasis or local irresectability. A further 15% dropout rate is expected due to perioperative complications that prevent adjuvant treatment in the perioperative group. A 30% dropout rate is expected in the adjuvant-only group due to disease progress, local irresectability, or perioperative complications. Thus, the total sample size is 166 (2 × 83) patients.

Demographic and baseline characteristics will be displayed separately by treatment groups using appropriate descriptive statistics. The primary objective, DFS rate at 18 months, will be evaluated by a one-sample log-rank test in each group on an intention-to-treat basis. The significance level will be set to 5% in each group. Because of the independency of both study arms there is no need for adjustment for multiple testing. As an explorative effect estimate, we will report the hazard ratio (with its corresponding 95% confidence interval) from a parallel Cox regression model by using various explaining variables including treatment group. It should be noted that this analysis uses the information from the whole-time course, the 18 months DFS rates given above were only used for the sample size calculation. However, for clinical reasons, we will also report DFS rates at 36 months. The analysis will be performed on an intention-to-treat basis.

It is important to mention, that the randomization between the two experimental arms is eminent to get two comparable patient groups and to investigate, which group and hereby which therapeutic concept (perioperative vs. adjuvant), compared to the fixed survival probability of 38% at 18 months, achieved the better outcome. No proof of superiority of one of the two treatment groups can be demonstrated with this study design because of low power, but this was not intended by the design of this phase II study.

Analyses for secondary objectives are considered purely exploratory and are given as risk or mean differences for binary and continuous responses, or as hazard ratios for survival outcome, all of them accompanied by the respective 95% confidence intervals. There will be no interim analysis on efficacy or subgroup analyses. Regarding safety, frequencies of SAEs and SUSARs will be reviewed regularly by the Data and Safety Monitoring Board. The final analysis on safety will include a comparison of risk differences between groups.

### Quality of life assessment

For measurement of Health related quality of life (HRQL) the EORTC QLQ-C30 version 3.0 will be used. The EORTC QLQ-C30 questionnaire is a validated, cancer-specific instrument designed for prospective clinical trials. Within the questionnaire five functions (physical, role, cognitive, emotional, and social), nine symptoms (fatigue, pain, nausea and vomiting, dyspnea, loss of appetite, insomnia, constipation, diarrhea and financial difficulties) and the global health status/quality of life (GBH/QoL) are assessed [32]. However used as a standard in EORTC studies, the QLQ-C30 lacks some quality of life dimensions for specific cancers. In line with that as certain module for pancreatic cancer (QLQ-PAN26) has been developed. The pancreatic cancer module is intended for patients at all disease stages. Within the module 26 items related to disease symptoms, treatment side effects and emotional issues specific to pancreatic cancer were recorded [33]. For detecting anxiety and depression, which are the most common co-morbidities of physical illness, the HADS-D questionnaire (Hospital Anxiety and Depression Scale – German version) is used. It provides a well-known measuring instrument with proven psychometric quality criteria [34, 35].

HRQL should be assessed at following time points:

- At baseline, within 7 days prior to randomization
- Prior CT-scan after 2 cycles of chemotherapy (Arm A)
- The day before surgery (or prior surgery within 3 days)
- After surgery within 4 weeks (week 3–4) prior CT-scan
- Before the beginning of each cycle of systemic therapy,
- At end of treatment visit about 4 weeks (+/− 7 days) after the last application dose of the study drugs
- During the follow up, every 3 months
- Quality of life assessment should be performed even when chemotherapy cannot be given at the beginning of a cycle e.g. due to toxicity reasons.

### Translational research

This trial provides the unique opportunity in pancreatic cancer to obtain material prior to and after surgery for biomarker analysis in correlation with outcome. We will perform pharmacogenomic candidate gene analysis of hENT1, CDA, DCK and 5’nucleotidase in both arms.

Core biopsies and specimens from the resected tumors will be analyzed by immunohistochemistry with respect to the activation of the hedgehog pathway, the Notch pathway, NFκB in the tumor and the stroma cells as well as CD3, CD40 and hENT1 immunoreactivity.

Exome sequencing provides a means of addressing the complexity and heterogeneity of the molecular mechanisms governing tumorigenesis and the underlying individual clinical response to the cytotoxic agents used. We hypothesize that exome sequencing of microdissected tumor cells will identify important biologic differences between tumors responding to cytotoxic chemotherapy compared to those not responding to the treatment and thereby provide potential predictive markers.

Consequently, we will collect preoperative and postoperative tissue samples for exome sequencing of best vs. worst responders. The best vs. worst responders will be assessed by a panel of independent pathologists establishing the regression grading.

Exome sequencing will primarily be done from material obtained by microdissection from the surgical specimens. Differences in gene expression will be verified in the material obtained preoperatively (core biopsies). In addition, we will analyze the core biopsies from tumors progressing in the neoadjuvant setting.

We will perform exome sequencing also in the control group in tumors with no signs of tumor regression compared to tumors with a high “spontaneous” regression. Sequencing results will be analyzed bioinformatically to separate the “spontaneous” regression effects from those associated with the preoperative treatment.

In parallel, blood samples (for each time point 5 × 7.5 ml EDTA-plasma) will be taken in the perioperative arm prior to treatment, prior to the beginning of cycle 2 and after completion of the neoadjuvant treatment prior to tumor resection, immediately after surgery and prior to the beginning of each new cycle of the adjuvant chemotherapy. In the adjuvant arm blood samples (for each time point 5 × 7.5 ml EDTA-plasma) will be taken prior to surgery, immediately after surgery and prior to the beginning of each new cycle of the adjuvant chemotherapy. Tumor DNA (ctDNA) will be extracted from blood samples and analyzed by targeted genotyping. In parallel, we will perform sequencing of patients’ lymphocytes to exclude artifacts due to prevailing germ line alterations.

Mutation profiles obtained from tissue and blood will be compared to evaluate whether tumor DNA analysis from blood yields a pattern comparable to tumor tissue and could be used to establish “easy to obtain” prognostic and predictive markers for gemcitabine and nab-paclitaxel. Additionally, miRNA analysis will be performed at a later stage, but material (blood) is collected and deposited in an appropriate biobank.

### Ethical aspects, trial registration

The ethics committee of Ulm University approved the NEONAX-trial as leading ethics committee for all German sites. In addition, local ethics committees approved the participating sites. The trial is registered with ClinicalTrials.gov (NCT02047513) and the European Clinical Trials Database (2013–005559-34).

## Discussion

Surgery of PDAC has improved over the last decade and has a high standard with low rates of perioperative morbidity and mortality in expert centers. However, the majority of patients even with resectable PDAC will succumb to their disease due to early relapse, even after R0 surgery. Currently, the 5-year overall survival rate with optimal adjuvant chemotherapy is at best 29% [36]. Long term survival after PDAC resection is still exceptional with a ≥ 10 year survival rate after surgery of 3.9% [37]. Neoadjuvant treatment in PDAC could be a valid tool for down-sizing or even downstaging the tumor thereby improving R0 resection rates. It may also be a valid strategy to reduce the risk of early metastasis given that a large proportion of PDACs are likely to be metastatic even when they appear clearly resectable as determined by conventional imaging [4].

FOLFIRINOX and nab-paclitaxel/gemcitabine have improved the standard of care for patients with metastatic PDAC compared to gemcitabine monotherapy [20, 22].

The high efficacy and the better tolerability make the combination of nab-paclitaxel and gemcitabine an interesting regimen to examine its use in a perioperative setting with the ultimate aim to improve long term survival.

Several trials address the value of an intensified adjuvant treatment strategy. The phase III APACT trial (NCT01964430) compares gemcitabine vs. nab-paclitaxel/gemcitabine in the adjuvant setting. First results of this trial are likely to be reported in 2019. This will give us further insights into the effectiveness of nab-paclitaxel/gemcitabine in this setting and will be of importance for the NEONAX trial since one arm of this trial uses the same regimen for adjuvant treatment. Recently, data from the GI-PRODIGE24/CCTG PA.6 trial (NCT01526135) were presented at the 2018 ASCO annual meeting. This trial showed significant superiority of adjuvant mFOLFIRINOX compared to gemcitabine after surgery for PDAC. mFOLFIRINOX thereby can be regarded as a new standard of care for the adjuvant therapy of patients with resectable pancreatic cancer. Nevertheless, neoadjuvant or perioperative treatment has potentially several advantages compared to the adjuvant setting: A significant proportion of patients does not receive adjuvant chemotherapy due to perioperative complications or prolonged postoperative recovery. In contrast, more than 80% of patients can receive neoadjuvant chemotherapy. Furthermore, a higher dose intensity can be achieved in the preoperative as compared to the postoperative setting [38] providing potentially a more efficacious treatment and the effect of a given chemotherapy on tumor regression can be directly evaluated. Ideally, tumor size is reduced and the percentage of true R0 resections increases by perioperative nab-paclitaxel/gemcitabine which is expected to result in better outcome. Furthermore, the preoperative part of the chemotherapy could indeed treat micrometastases and/or limit tumor seeding during surgery. Thus, the critical question addressed by the NEONAX trial is whether we can achieve a better systemic tumor control/reduce metastasis using nab-paclitaxel/gemcitabine in a perioperative or in an adjuvant setting, respectively. Furthermore, neoadjuvant chemotherapy may allow us to study the biology of a given tumor and may help to identify patients who indeed benefit from neoadjuvant treatment and/or surgery.

Tumor progress during intensified neoadjuvant chemotherapy is a potential concern. It occurs in up to 20% of cases, either locally or by the occurrence of metastases. However, progress during treatment with an intensified chemotherapy protocol is likely to be an indicator of a particularly poor tumor biology and suggests that these patients would not have benefitted from surgery due to an early relapse after surgery.

The translational research program of the NEONAX trial provides the unique opportunity to obtain material (tissue and liquid biopsies) prior to and after surgery and during systemic therapy for biomarker analysis and correlation with outcome. This give us the unique chance to identify subgroups of patients who really benefit and those who do not benefit from perioperative systemic therapy in order to personalize PDAC treatment.

There are several recruiting trials worldwide addressing the status of a neoadjuvant/perioperative systemic treatment in resectable PDAC, e.g. the SWOG S1505 trial (NCT02562716, neoadjuvant FOLFIRINOX vs. gemcitabine/nab-paclitaxel in resectable pancreatic cancer), the NEOPAC study (NCT01314027, neoadjuvant gemcitabine/oxaliplatin plus adjuvant gemcitabine vs. adjuvant gemcitabine in resectable pancreatic cancer) [39], the NEPAFOX trial (NCT02172976, neoadjuvant/adjuvant FOLFIRINOX vs. adjuvant gemcitabine in resectable pancreatic cancer) but the NEONAX-trial is at the moment the biggest neoadjuvant trial with an extended translational program in resectable pancreatic cancer worldwide and has started recruitment in Q1/2015 at 26 German university hospitals or high-volume centers for PDAC surgery.

## Additional Files

Additional File 1: SPIRIT 2013 checklist. (DOC 123 kb)

Additional File 2: Frequency and scope of study visits. (DOCX 59 kb)

## Abbreviations

(m)DFS (median): disease free survival
(m)OS (median): overall survival
(m)PFS (median): progression free survival
AIO: Arbeitsgemeinschaft Internistische Onkologie
BSC: best supportive care
CBC: complete blood count
CT: Computed Tomography
CTC: common toxicity criteria
CTCAE: Common Terminology Criteria for Adverse Events
ctDNA: circulating tumor DNA
ECG: electrocardiogram
ECOG: Eastern Cooperative Oncology Group
EORTC: European Organization for Research and Treatment of Cancer
FOLFIRINOX: fluorouracil leucovorine, irinotecan, oxaliplatin
GBH: global health status
G-CSF: granulocyte-colony stimulating factor
HADS-D: Hospital Anxiety and Depression Scale
HR: hazard ratio
HRQL: Health related quality of life
ITT: intention to treat
MRI: Magnetic Resonance Imaging
Nab-Paclitaxel: nano albumin bound Paclitaxel
NCI: National Cancer Institute
PDAC: pancreatic ductal adenocarcinoma
QLQ-C30: quality of life questionnaire-core 30
QoL: quality of life
RECIST: Response Evaluation Criteria in Solid Tumors
TUDD: time until definitive deterioration
ULN: upper limit of normal
