# Supplementary material for: Neoadjuvant plus adjuvant or only adjuvant nab-paclitaxel plus gemcitabine for resectable pancreatic cancer - the NEONAX trial (AIO-PAK-0313), a prospective, randomized, controlled, phase II study of the AIO pancreatic cancer group
Source: BMC Cancer. 2018 Dec 29;18:1298. doi: 10.1186/s12885-018-5183-y (PMC6311014; doi:10.1186/s12885-018-5183-y)
Supplement: Supplementary file 2 — Frequency and scope of study visits. (DOCX 59 kb) [file 12885_2018_5183_MOESM2_ESM.docx]

Spirit diagram

**Neoadjuvant plus adjuvant or only adjuvant nab-Paclitaxel plus Gemcitabine for resectable pancreatic cancer**

**- The NEONAX trial (AIO-PAK-0313), a prospective, randomized, controlled, phase II study of the AIO Pancreatic Cancer Group**

## Frequency and scope of study visits – Arm A – perioperative arm

| **Required assessments** | **Screening** | **Neoadjuvant treatment** | | | | | | **Surgery** | | | **Adjuvant treatment** | | | | | | | | | | | | **EOT visit** | **Follow-up period** |
| --- | --- | --- | --- | --- | --- | --- | --- | --- | --- | --- | --- | --- | --- | --- | --- | --- | --- | --- | --- | --- | --- | --- | --- | --- |
|  | Day -28 to -1 | Cycle I, d1 | Cycle I, d8 | Cycle I, d15 | Cycle II, d1 | Cycle II, d8 | Cycle II, d15 | Staging and Break for up to 3 weeks | Tumor resection | Break for up to 12 weeks | Cycle III, d1 | Cycle III, d8 | Cycle III, d15 | Cycle IV, d1 | Cycle IV, d8 | Cycle IV, d15 | Cycle V, d1 | Cycle V, d8 | Cycle V, d15 | Cycle VI, d1 | Cycle VI, d8 | Cycle VI, d15 | about d28 after last study drug |  |
| Signed Informed Consent | X |  |  |  |  |  |  |  |  |  |  |  |  |  |  |  |  |  |  |  |  |  |  |  |
| Demography | X |  |  |  |  |  |  |  |  |  |  |  |  |  |  |  |  |  |  |  |  |  |  |  |
| Medical history | X |  |  |  |  |  |  |  |  |  |  |  |  |  |  |  |  |  |  |  |  |  |  |  |
| Quality of life^f^ | X | X |  |  | X |  |  | X |  | X | X |  |  | X |  |  | X |  |  | X |  |  | X | X |
| Proof of pancreatic adenocarcinoma (Core biopsy)^a^ | X |  |  |  |  |  |  |  |  |  |  |  |  |  |  |  |  |  |  |  |  |  |  |  |
| Physical examination^b^ | X | X | X | X | X | X | X | X |  |  | X | X | X | X | X | X | X | X | X | X | X | X | X | X |
| Eligibility/ Enrollment | X |  |  |  |  |  |  |  |  |  |  |  |  |  |  |  |  |  |  |  |  |  |  |  |
| ECG | X |  |  |  |  |  |  |  |  |  |  |  |  | X |  |  |  |  |  |  |  |  | X |  |
| Echocardiography | X |  |  |  |  |  |  |  |  |  |  |  |  | X |  |  |  |  |  |  |  |  | X |  |
| End. Ultrasound Elasticity Imaging (optional) | X |  |  |  |  |  |  | X |  |  |  |  |  |  |  |  |  |  |  |  |  |  |  |  |
| Serum pregnancy test^c^ | X |  |  |  |  |  |  |  |  | X |  |  |  |  |  |  |  |  |  |  |  |  |  |  |
| Haematology and Clinical chemistry^d^ | X | X | X | X | X | X | X | X |  |  | X | X | X | X | X | X | X | X | X | X | X | X | X |  |
| Adverse events/ Toxicities | X | X | X | X | X | X | X | X | X^g^ | X^g^ | X | X | X | X | X | X | X | X | X | X | X | X | X |  |
| Concomitant medications | X | X | X | X | X | X | X | X |  |  | X | X | X | X | X | X | X | X | X | X | X | X | X |  |
| Tumor assessment^e^ | X |  |  |  |  |  |  | X |  | X |  |  |  |  |  |  |  |  |  |  |  |  | X | X |
| Ca19-9, CEA | X | X |  |  | X |  |  | X |  |  | X |  |  | X |  |  | X |  |  | X |  |  | X | X |
| blood for translational research |  | X |  |  | X |  |  | X |  | X | X |  |  | X |  |  | X |  |  | X |  |  | X |  |
| Survival status | X | X | X | X | X | X | X | X | X | X | X | X | X | X | X | X | X | X | X | X | X | X | X | X |
| Chemotherapy |  | X | X | X | X | X | X |  |  |  | X | X | X | X | X | X | X | X | X | X | X | X |  |  |

a Histological proof of the pancreatic adenocarcinoma. A new core biopsy is not required, if the lesion has been biopsied previously with a 19 G needle. Alternatively tissue samples can be obtained via laparoscopic surgery in particular cases

b This includes height (only at screening), weight, ECOG performance status, vital signs (blood pressure, heart rate, respiratory rate, oral body temperature), clinical signs of heart failure (e.g. peripheral edema, pleural effusions, dyspnea) or clinical signs of progressive polyneuropathy (e.g. featuring weakness, numbness, pins-and-needles and burning pain beginning in hands and feet), short-questionnaire “symptoms of polyneuropathy”

c Only for women with childbearing potential within 7 days of the application of chemotherapy (and if indicated in the opinion of the investigator)

d Hemoglobin, hematocrit, platelet count, white blood count including differential blood count, sodium, potassium, chloride, calcium, magnesium, phosphate, AST, ALT, total bilirubin, alkaline phosphatase, uric acid, total protein, albumin, LDH, creatinine, blood urea nitrogen, PT (must be given as INR), PTT, CRP

e Abdominal CT or MRI and chest x-ray for screening and during treatment, follow-up period: abdominal CT/MRI and chest x-ray every 3 months routinely for 3 years, than abdominal ultrasound every 3 months (if suspicious for relapse: CT/MRI) and abdominal CT/MRI and chest x-ray every 6 months, as an alternative to chest -ray, thoracic CT can be performed at the discretion of the center (recommended)

f Quality of life assessment should be performed even when the chemotherapy cannot be applied at the beginning of a cycle e.g. due to toxicity reasons

g see Assessments during surgery/resections

## Frequency and scope of study visits – Arm B– adjuvant arm

| **Required assessments** | **Screening** | **Surgery** | | | **Adjuvant treatment** | | | | | | | | | | | | | | | | | | **EOT visit** | **Follow-up period** |
| --- | --- | --- | --- | --- | --- | --- | --- | --- | --- | --- | --- | --- | --- | --- | --- | --- | --- | --- | --- | --- | --- | --- | --- | --- |
|  | Day -28 to -1 | Day 0 to 14 | Tumor resection | Break for up to 12 weeks | Cycle I, d1 | Cycle I, d8 | Cycle I, d15 | Cycle II, d1 | Cycle II, d8 | Cycle II, d15 | Cycle III, d1 | Cycle III, d8 | Cycle III, d15 | Cycle IV, d1 | Cycle IV, d8 | Cycle IV, d15 | Cycle V, d1 | Cycle V, d8 | Cycle V, d15 | Cycle VI, d1 | Cycle VI, d8 | Cycle VI, d15 | about d28 after last study drug |  |
| Signed Informed Consent | X |  |  |  |  |  |  |  |  |  |  |  |  |  |  |  |  |  |  |  |  |  |  |  |
| Demography | X |  |  |  |  |  |  |  |  |  |  |  |  |  |  |  |  |  |  |  |  |  |  |  |
| Medical history | X |  |  |  |  |  |  |  |  |  |  |  |  |  |  |  |  |  |  |  |  |  |  |  |
| Quality of life^f^ | X | X |  | X | X |  |  | X |  |  | X |  |  | X |  |  | X |  |  | X |  |  | X | X |
| Proof of PDAC (Core biopsy) | X |  |  |  |  |  |  |  |  |  |  |  |  |  |  |  |  |  |  |  |  |  |  |  |
| Physical examination^b^ | X |  |  |  | X | X | X | X | X | X | X | X | X | X | X | X | X | X | X | X | X | X | X | X |
| Eligibility/ Enrollment | X |  |  |  |  |  |  |  |  |  |  |  |  |  |  |  |  |  |  |  |  |  |  |  |
| ECG | X |  |  |  |  |  |  |  |  |  |  |  |  | X |  |  |  |  |  |  |  |  | X |  |
| Echocardiography | X |  |  |  |  |  |  |  |  |  |  |  |  | X |  |  |  |  |  |  |  |  | X |  |
| End. Ultrasound Elasticity Imaging (optional) | X |  |  |  |  |  |  |  |  |  |  |  |  |  |  |  |  |  |  |  |  |  |  |  |
| Serum pregnancy test^c^ | X |  |  | X |  |  |  |  |  |  |  |  |  |  |  |  |  |  |  |  |  |  |  |  |
| Haematology and Clinical chemistry^d^ | X | X |  |  | X | X | X | X | X | X | X | X | X | X | X | X | X | X | X | X | X | X | X |  |
| Adverse events/ Toxicities | X | X | X^g^ | X^g^ | X | X | X | X | X | X | X | X | X | X | X | X | X | X | X | X | X | X | X |  |
| Concomitant medications | X |  |  |  | X | X | X | X | X | X | X | X | X | X | X | X | X | X | X | X | X | X | X |  |
| Tumor assessment^e^ | X |  |  | X |  |  |  |  |  |  |  |  |  |  |  |  |  |  |  |  |  |  | X | X |
| Ca19-9, CEA | X |  |  |  | X |  |  | X |  |  | X |  |  | X |  |  | X |  |  | X |  |  | X | X |
| blood for translational research |  | X |  | X | X |  |  | X |  |  | X |  |  | X |  |  | X |  |  | X |  |  | X |  |
| Survival status | X | X | X | X | X | X | X | X | X | X | X | X | X | X | X | X | X | X | X | X | X | X | X | X |
| Chemotherapy |  |  |  |  | X | X | X | X | X | X | X | X | X | X | X | X | X | X | X | X | X | X |  |  |

a Histological proof of the pancreatic adenocarcinoma.

b This includes height (only at screening), weight, ECOG performance status, vital signs (blood pressure, heart rate, respiratory rate, oral body temperature), clinical signs of heart failure (e.g. peripheral edema, pleural effusions, dyspnea) or clinical signs of progressive polyneuropathy (e.g. featuring weakness, numbness, pins-and-needles and burning pain beginning in hands and feet), • short-questionnaire “symptoms of polyneuropathy”

c Only for women with childbearing potential within 7 days of the application of chemotherapy (and if indicated in the opinion of the investigator)

d Hemoglobin, hematocrit, platelet count, white blood count including differential blood count, sodium, potassium, chloride, calcium, magnesium, phosphate, AST, ALT, total bilirubin, alkaline phosphatase, uric acid, total protein, albumin, LDH, creatinine, blood urea nitrogen, PT (must be given as INR), PTT, CRP

e Abdominal CT or MRI and chest x-ray for screening and during treatment, follow-up period: abdominal CT/MRI and chest x-ray every 3 months routinely for 3 years, than abdominal ultrasound every 3 months (if suspicious for relapse: CT/MRI) and abdominal CT/MRI and chest x-ray every 6 months, as an alternative to chest -ray, thoracic CT can be performed at the discretion of the center (recommended)

f Quality of life assessment should be performed even when the chemotherapy cannot be applied at the beginning of a cycle e.g. due to toxicity reasons

g see Assessments during surgery/resections
